# Whole‐exome sequencing reveals a long‐term decline in effective population size of red spruce (*Picea rubens*)

**DOI:** 10.1111/eva.12985

**Published:** 2020-05-22

**Authors:** Thibaut Capblancq, John R. Butnor, Sonia Deyoung, Ethan Thibault, Helena Munson, David M. Nelson, Matthew C. Fitzpatrick, Stephen R. Keller

**Affiliations:** ^1^ Department of Plant Biology University of Vermont Burlington VT USA; ^2^ USDA Forest Service Southern Research Station University of Vermont Burlington VT USA; ^3^ Appalachian Laboratory University of Maryland Center for Environmental Science Frostburg MD USA

**Keywords:** demographic history, forest management, genomics, *Picea rubens*

## Abstract

Understanding the factors influencing the current distribution of genetic diversity across a species range is one of the main questions of evolutionary biology, especially given the increasing threat to biodiversity posed by climate change. Historical demographic processes such as population expansion or bottlenecks and decline are known to exert a predominant influence on past and current levels of genetic diversity, and revealing this demo‐genetic history can have immediate conservation implications. We used a whole‐exome capture sequencing approach to analyze polymorphism across the gene space of red spruce (*Picea rubens* Sarg.), an endemic and emblematic tree species of eastern North America high‐elevation forests that are facing the combined threat of global warming and increasing human activities. We sampled a total of 340 individuals, including populations from the current core of the range in northeastern USA and southeastern Canada and from the southern portions of its range along the Appalachian Mountains, where populations occur as highly fragmented mountaintop “sky islands.” Exome capture baits were designed from the closely relative white spruce (*P. glauca* Voss) transcriptome, and sequencing successfully captured most regions on or near our target genes, resulting in the generation of a new and expansive genomic resource for studying standing genetic variation in red spruce applicable to its conservation. Our results, based on over 2 million exome‐derived variants, indicate that red spruce is structured into three distinct ancestry groups that occupy different geographic regions of its highly fragmented range. Moreover, these groups show small *N_e_*, with a temporal history of sustained population decline that has been ongoing for thousands (or even hundreds of thousands) of years. These results demonstrate the broad potential of genomic studies for revealing details of the demographic history that can inform management and conservation efforts of nonmodel species with active restoration programs, such as red spruce.

## 1 INTRODUCTION

The demographic events that have punctuated the evolutionary history of a species are among the most influential processes explaining the current distribution of genetic diversity on the landscape (Ellegren & Galtier, 2016; Hewitt, 1996; Keinan & Andrew, 2012). Such events, including population bottlenecks or growth, range expansion or contraction, migration, and population splitting have tremendous effects on allele frequency distributions, both within and among populations (Hoban et al., 2010; Nei, Maruyama, & Chakraborty, 1975). Of particular importance are changes in the effective population size (*N*_e_) that influence the magnitude of genetic drift within a population and that lead to variation in the rate of allele loss and fixation (Ellegren & Galtier, 2016; Foll & Gaggiotti, 2006). For instance, a declining population experiences an increased level of genetic drift, which at very low *N*_e_ can drive a rapid decrease in genetic diversity even though a declining population is also more exposed to the deleterious effects of inbreeding depression (Frankham, 2005). In contrast, an expanding population is predicted to lose fewer alleles through drift while also accumulating more genetic variation through de novo mutation, which is the raw material for selection (Barrett & Schluter, 2008; de Lafontaine & Bousquet, 2017). In this context, high levels of intraspecific genetic variation are positively associated with a population's long‐term persistence and ability to adapt to changing environments (Jump, Marchant, & Peñuelas, 2009). Investigating the effects of past demographic history is therefore of particular importance when evaluating current population dynamics, for example, when trying to estimate population viability or long‐term capacity for evolutionary change. The importance of understanding this demo‐genetic history is even more critical now that biodiversity is facing threats from global climate change and increasing human activities (Lavergne, Mouquet, Thuiller, & Ronce, 2010). However, such information is lacking for many species of conservation concern.

Red spruce (*Picea rubens* Sarg.) is a coniferous tree species endemic to eastern North American high‐elevation forests. Current populations of red spruce are mainly distributed in mountainous locations across the northeastern USA and the cool maritime climates of eastern Canada. However, this species also occurs in fragmented patches at high elevations along the Appalachian Mountains in the Mid‐Atlantic and southeastern USA (Adams & Stephenson, 2019). Red spruce is a keystone species of the high‐altitude Appalachians forests, which provide refuge for numerous endemic and emblematic animal and plant species (Diggins & Ford, 2017; Rentch, Ford, Schuler, & Palmer, 2016), and is also economically valued for the high quality of its wood, particularly in the manufacturing of musical instruments (Dumais & Prévost, 2007).

Throughout its range, red spruce populations have experienced significant human‐caused decline from logging and fire in the late 1800 to early 1900s, and more recently from atmospheric pollution leading to acid rain (Mathias & Thomas, 2018; Rentch et al., 2016). These events likely impacted genetic diversity and in some cases may have caused significant selection on standing genetic variation for tolerance to atmospheric pollution (Bashalkhanov, Eckert, & Rajora, 2013). The decline of red spruce, by as much as 95% of its original areal extent in the highly fragmented southern portion of its range (Rentch & Schuler, 2009), has become a major conservation focus among resource managers and restoration ecologists, which has led to the formation of multipartner cooperatives aimed at restoring functional red spruce ecosystems via large‐scale restoration plantings and relevant silvicultural practices (e.g., the Central and Southern Appalachian Spruce Restoration Initiatives; CASRI and SASRI, respectively). Since the passage of the Clean Air Act of 1970 and the reduction in atmospheric pollution, red spruce has shown some recovery in terms of growth of existing trees and new recruitment (Kosiba, Schaberg, Rayback, & Hawley, 2018; Mathias & Thomas, 2018; Verrico, Weiland, Perkins, Beckage, & Keller, 2019), encouraging the ongoing conservation efforts.

While it is clear that red spruce has experienced dramatic short‐term demographic decline as the result of human activities and possibly demographic recovery in some portions of its range, its longer‐term demographic history of population stability or growth/decline over evolutionary timescales is largely unknown. Genetic diversity within red spruce is thought to be quite low (Rajora, Mosseler, & Major, 2000), possibly indicating a historically low *N*_e_ that may reflect an ongoing bottleneck that arose during its speciation event from black spruce (*Picea mariana*), its closest relative. Further, while there is some indication that red spruce populations show differences in genetic ancestry corresponding to different regions within its highly fragmented range (Bashalkhanov et al., 2013), a clear picture of this genetic substructure and its correspondence to variability in the timing or magnitude of *N*_e_ changes is lacking. Understanding this demo‐genetic history has immediate conservation implications, such as assessing where mitigation of *N*_e_ reductions may be most needed based on historical trajectories in different part of the species' range (Flanagan, Forester, Latch, Aitken, & Hoban, 2018; Leroy et al., 2018).

One key limitation affecting most studies on red spruce to date has been the lack of dense and genome‐wide polymorphism data, which is essential to obtain precise estimates of population genetic demographic parameters when diversity levels are inherently low. Thus, a critical next step toward integrating genomics into conservation strategies for this system requires the establishment of a dense genomic dataset on a range‐wide sample to enable a detailed understanding of local variability in genetic diversity and temporal trends in *N*_e_.

To accomplish these goals, we used a whole‐exome capture approach to focus sequencing efforts on the protein‐coding regions of the massive 20 + Gb spruce genome (Birol et al., 2013; Nystedt et al., 2013). Whole‐genome sequencing of conifer genomes for population‐level studies is not cost‐effective (Suren et al., 2016), but sequence capture is increasingly used for population‐level studies (Chen et al., 2019; Holliday, Zhou, Bawa, Zhang, & Oubida, 2016; Müller, Freund, Wildhagen, & Schmid, 2015; Zhou & Holliday, 2012) and has been successfully applied to several other spruce species including *P. abies* (Azaiez et al., 2018; Chen et al., 2019), *P. glauca* and *P. engelmannii* (Rigault et al., 2011; Suren et al., 2016), and *P. mariana* (Lenz et al., 2017). By targeting just the exomic regions of the genome (~1% of the spruce genome (Suren et al., 2016)), we were able to genotype hundreds of individuals across the species' range sequenced at low coverage (~2–3x). We paired this sequencing strategy with computational advancements in genotype‐free population genomic methods designed for low‐coverage data (Korneliussen, Albrechtsen, & Nielsen, 2014; Nielsen, Paul, Albrechtsen, & Song, 2011; Vieira, Fumagalli, Albrechtsen, & Nielsen, 2013) to investigate the current and historic genetic diversity of red spruce's populations.

Specifically, in this study we address the following objectives: (a) to determine the effectiveness of whole‐exome capture sequencing at low coverage to investigate genome‐wide patterns of population genetic diversity; (b) to investigate signatures left by past demographic events on the current genetic structure across red spruce's range; and (c) to estimate regional differences in *N*_e_ over time to assess the demographic trajectory of populations. We highlight how pairing low‐coverage capture sequencing with genotype‐free analytical methods from population genomics can yield novel insights into the demo‐genetic history of nonmodel species with large genomes. Further, we demonstrate broad potential for integrating these findings into management and conservation efforts of species with active restoration programs, such as red spruce.

## 2 MATERIALS & METHODS

### 2.1 Sampling design and DNA material

**Figure 1:**
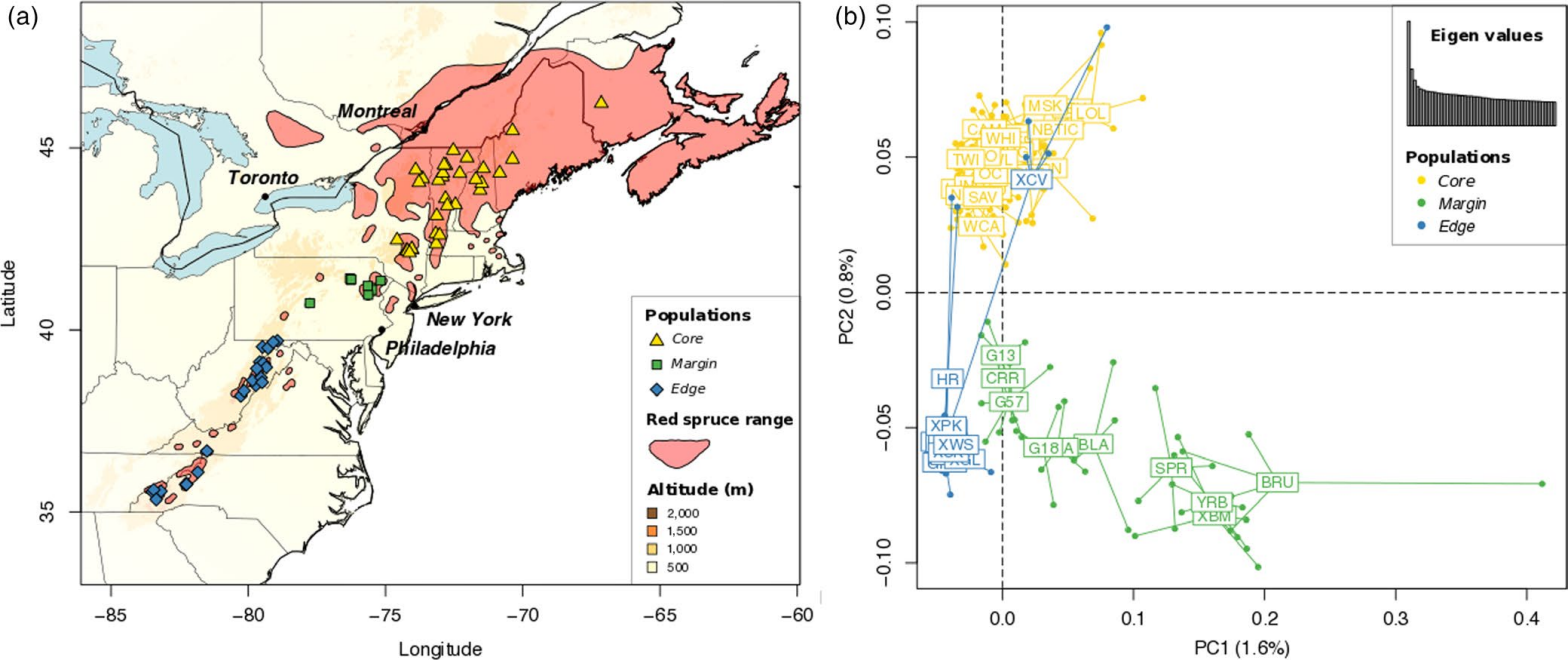
Geographic distribution of red spruce in eastern North America, including the sampled populations in the Core, Margin, and Edge regions (a). Genetic structure among red spruce populations revealed by the two first axes (PCs) of a genetic principal component analysis (PCA), performed using the genotype likelihoods for 2.18 M SNPs (b). A barplot of the 50 first eigenvalues shows the importance of PC1 and PC2 in comparison with the following axes

We sampled 65 populations and 340 individuals of red spruce across the entirety of its range in eastern North America (Table S1). Samples were distributed geographically to include the main portion of red spruce's range in northeastern USA and southeastern Canada, where red spruce is abundant and highly connected, as well as in the southern portions of its range in Pennsylvania and southeastern states (Maryland, West Virginia, Virginia, North Carolina, Tennessee), where populations occur as isolated mountaintop “sky islands” (Figure 1a). From each individual tree, we sampled fresh needle tissue that was transported back to the laboratory and stored at −80°C until processing. Approximately 50 mg of fresh frozen tissue per individual was then flash‐frozen in liquid nitrogen, and ground to a fine powder using a Qiagen TissueLyser II, and whole‐genomic DNA was extracted using the Qiagen DNeasy 96 Plant Kit. The quality of extracted DNA was assessed on 2% agarose gels, and DNA quantity was determined fluorometrically using the QuANT‐IT dsDNA assay kit (Thermo‐Fisher Scientific) read on a 96‐well plate reader (BioTek Instruments).

### 2.2 Whole‐exome sequence capture

*Bait design—*Our aim in the bait design was to include exomic regions from as many genes as possible, together with upstream and downstream regulatory regions and intergenic sequences between nearby genes. To design the capture baits, we used two reference transcriptomes of white spruce (*Picea glauca*), a closely related species of red spruce (Lockwood et al., 2013), previously assembled by Rigault et al., (2011) and Yeaman et al., (2014). Unigene sequences from these assembled transcriptomes were mapped to the white spruce genome (Warren et al.., 2015) using GMAP (Wu & Watanabe, 2005), and regions that mapped uniquely to the reference were considered to represent exomic regions and were subsequently used for designing probes. A total of 80,000 120 bp probes were designed, including 75,732 probes within or overlapping exomic regions, and an additional 4,268 probes in intergenic regions. Each probe was required to represent a single blast hit to the genome of at least 90 bp long and 85% identity, covering 38,570 unigenes. Probes were designed and synthesized at RAPiD Genomics (Florida, USA).

Capture*—*From extracted DNA, libraries were made by random mechanical shearing of DNA (250 ng −1µg) to an average size of 400 bp followed by end‐repair reaction, ligation of an adenine residue to the 3'‐end of the blunt‐end fragments to allow the ligation of barcoded adapters, and PCR amplification of the library. SureSelect probes (Agilent Technologies) were used for solution‐based targeted enrichment of pools of 16 libraries, following the SureSelect^xt^ Target Enrichment System for Illumina Paired‐End Multiplexed Sequencing Library protocol. Libraries were sequenced on an Illumina HiSeq X to generate paired‐end 150‐bp reads.

### 2.3 Sequence alignment and genotype likelihood estimation

Read mapping—Libraries were demultiplexed, clipped of Illumina adapter sequences, and trimmed of low‐quality flanking sequence (Q < 20) using a sliding window of 6 bp using Trimmomatic (Bolger, Lohse, & Usadel, 2014).

We used the most recent version of the nonhybrid *P. glauca* reference genome (WS7111; Birol et al., 2013) as source of reference genome sequence. However, as the full reference genome was too large (26.9 Gbp) for some downstream tools, we reduced the reference by retaining only those genomic contigs that had probes aligning to them. This reduced reference included 2.5 Gbp distributed across 27,479 of the 3,353,683 WS711 scaffolds. Mapping the spruce transcriptome assemblies produced by Rigault et al. (2011) and Suren et al. (2016) against this reduced reference with GMAP, we found that at >34.9 Mbp of the reduced reference consisted of exomic regions, very close to the estimated size of the *Picea* exome (Rigault et al., 2011).

To select a mapping procedure amenable to our study design (i.e., 150 bp paired‐end reads, and a large and highly fragmented reference genome), we compared the performance of three commonly used aligners: BWA (Li & Durbin, 2009), Stampy (Lunter & Goodson, 2011), and NextGenMap (Sedlazeck, Rescheneder, & Von Haeseler, 2013). We mapped the reads of 92 randomly selected individuals to the reduced reference using either the BWA‐MEM algorithm, marking short split hits as secondary (“‐M” option); the Stampy pipeline, by remapping the BAM files obtained with BWA‐MEM (“‐bamkeepgoodreads” option); or the NextGenMap program with default options. We assessed mapping success as the number of mapped reads, the number of properly paired mapped reads, and the number of reads mapped with a mapQ score higher than 10, 20, and 30. For our data, BWA proved more effective than NextGenMap, with the percentage of reads mapped with high mapQ two times greater than NextGenMap (Figure S1). Mapping quality was very similar between BWA and Stampy; however, BWA was tremendously faster, which led us to prefer BWA over Stampy for this study.

Final mapping was then performed with the program BWA, using the BWA‐MEM algorithm. The SAM files resulting from the aligned reads were converted to BAM files using SAMtools (Li et al., 2009). PCR duplicates were removed using the *“markdup”* function in sambamba‐0.6.8 (Tarasov, Vilella, Cuppen, Nijman, & Prins, 2017). One individual (YRB_01) was removed from further analyses due to more than 50% of PCR duplicates. The filtered BAM files were then sorted and indexed using SAMtools.

Genotype likelihood estimation*—*Low‐coverage sequencing (i.e., less than 5 reads per site and individual) greatly increases the probability that only one of the two chromosomes of a diploid individual gets sampled during sequencing, leading to uncertainty in genotype calling (Nielsen et al., 2011). To take this uncertainty into account for downstream analyses, while still taking full advantage of the breadth of sequencing coverage across sites and individuals, we used the program ANGSD (Analysis of Next Generation Sequencing Data) (Korneliussen et al., 2014). This program does not hard call genotypes but instead calculates a genotype likelihood for each polymorphic site, based on the depth of aligned reads and the associated mapping and sequencing quality scores.

Genotype likelihoods were estimated using the SAMtools genotype likelihood model, only using reads having unique best hits (“‐uniqueOnly 1”), setting a minimum MapQ score to keep a read to 20 (“‐minMapQ 20”), a min nucleotide Q score to consider a site to 20 (“‐minQ 20”), a minimum number of 2 individuals with coverage to keep a site (“‐minInd 2”), a maximum of 17 reads to estimate genotype likelihood for one individual (“‐setMaxDepthInd 17”), a minimum number of 15 reads across the complete sampling to estimate genotype likelihoods for a site (“‐setMinDepth 15”), keeping only biallelic sites (“‐skipTriallelic 1”), and performing the base alignment quality (BAQ: Phred‐scaled probability of a read base being misaligned; Li, 2011) as in SAMtools (“‐baq 1”). The resulting genotype likelihoods were then used for downstream analyses.

We functionally annotated variants based on the published annotations for the Norway spruce (*Picea abies*) genome, available from congenie.org (Nystedt et al., 2013). To do so, we conducted local BLAST searches of a custom database using the command line version of *blastn* (Camacho et al., 2009) to map the coordinates of the white spruce scaffolds containing our mapped exome reads against the Norway spruce genome (Nystedt et al., 2013). Using the mapped positions of the red spruce SNPs within the Norway spruce reference, we then used SNPeff (Cingolani et al., 2012) to annotate variants to functional class (upstream and downstream, introns, synonymous, nonsynonymous, intronic, or intergenic sites) based on the Norway spruce genome annotation.

### 2.4 Genetic structure and population genomic diversity

We assessed the genetic structure of red spruce through principal component analysis (PCA) from the genotype likelihoods produced with ANGSD and keeping only sites at least 500 bp apart from each other to avoid linkage disequilibrium (see Results section: *3.4*. Linkage disequilibrium and nucleotide diversity, for the estimation of LD in the full dataset). To perform the PCA, we used the ‐doCov option of ANGSD to produce a matrix of genetic covariance among all 339 individuals, which we used to find the eigenvalues of the matrix and plot the eigenvectors based on individual loadings. We also estimated pairwise population fixation index (*F*_ST_) values between each pair of the three regional subpopulations (hereafter, “regions”). Regions were designated a posteriori to investigate potential differences in regional genomic diversity and effective population size dynamics based on clear clustering of individuals in the PCA (See Results section: 4.3. Genetic differentiation). These genetic regions corresponded to three geographically distinct areas: the main spatially contiguous northern part of the range (hereafter called “Core,” 178 samples), the area of Pennsylvania where the main portion of the range begins to become fragmented (hereafter called “Margin,” 51 samples), and a low latitude highly fragmented trailing edge region in the southern Appalachians (hereafter called “Edge,” 110 samples). The *F*_ST_ values were estimated using the realSFS subprogram of ANGSD and from the SFS of each region, derived from the genotype likelihoods of the same sites used to perform the PCA (i.e., sites covered in all three regions and separated by at least 500 bp).

To characterize population genomic parameters of diversity, we estimated nucleotide diversity based on Watterson's theta (*θ*_w_) (Nei et al., 1975; Tajima, 1983), Tajima's *D* (Tajima, 1989), pairwise nucleotide diversity (π), and linkage disequilibrium (LD) across the exome using genotype likelihoods of all the sites (i.e., monomorphic and polymorphic). We estimated diversity parameters for each of the three regions using the same filtering options as described above. Site‐specific Watterson's theta (*θ*_w_), pairwise diversity (π), and Tajima's *D* were calculated using the program ANGSD and its function “*thetaStat*” (Thorfinn Sand Korneliussen, Moltke, Albrechtsen, & Nielsen, 2013) from the region‐specific genotype likelihood files. Pairwise LD was estimated using the program ngsLD (Fox, Wright, Fumagalli, & Vieira, 2019), restricting comparisons to between pairs of polymorphic sites within the same scaffold. To estimate the rate of LD decay across the red spruce exome, we fit a nonlinear decay model to the observed distribution of pairwise LD (*r* ^2^) as a function of physical distance (bp) based on the equation initially proposed by Hill and Weir (1988) and revised by Remington et al. (2001).

### 2.5 Inference of historic population dynamics

We used different complementary approaches to investigate the historic demography of population growth or decline in red spruce, the STAIRWAY PLOT method (Liu & Fu, 2015), a phylogenomic analysis conducted with TreeMix (Pickrell & Pritchard, 2012) and a likelihood approach implemented in Fastsimcoal2 (Excoffier, Dupanloup, Huerta‐Sánchez, Sousa, & Foll, 2013). For both STAIRWAY PLOT and Fastsimcoal2 approaches, we used the realSFS program from ANGSD to build a site frequency spectrum (SFS) from the genotype likelihoods for each of three regional groups defined by the genetic PCA (see Results). Sites covered in all three regions were used to compute the SFS, meaning that each SFS potentially included sites monomorphic for all three regions, sites monomorphic for one region but polymorphic in another, and sites polymorphic for two or three regions. A total sequence length of 44.2 Mb was used for creating the SFS, including monomorphic and polymorphic sites. The three regional SFS were then projected (rarified) down to 40 individuals to avoid missing genotypes and optimize the resolution (Gutenkunst, Hernandez, Williamson, & Bustamante, 2009). For TreeMix analysis, we inferred genotypes with ANGSD (‐doGeno 2) and estimated allele counts for each polymorphic site in each region.

We used the STAIRWAY PLOT method (Liu & Fu, 2015) to obtain an estimate of the effective population size (*N*_e_) across the evolutionary history of our sample. We converted from coalescent units to absolute units of population size and time assuming a generation time of 29 years (Wu, McCormick, & Busing, 1999) and a silent mutation rate of 2.0 × 10^–9^ per site per generation (Nystedt et al., 2013). Effective population sizes through time were estimated using the median of 200 bootstrap replicates, and the precision of the estimations was evaluated using 95% confidence intervals.

As a complement to the STAIRWAY PLOT method, we investigated the history of *N*_e_ in our sample using a multipopulation demographic model that allowed for divergence and migration among regions. To do so, we first used the program TreeMix (Pickrell & Pritchard, 2012) to infer the history of divergence among regions and the presence of potential migration events. A maximum‐likelihood phylogenetic tree was built for the three different regional pools of individuals, using 10 white spruce individuals as outgroup. The white spruce sequences were recovered from the NCBI (Suren et al., 2016) and mapped and treated the same way as the red spruce samples to infer genotypes for the same sites. The TreeMix maximum‐likelihood tree returned a scenario where the Core region split first, followed by a split between Edge and Margin, and evidence for several migration events (Figure S3). We then used Fastsimcoal2 (Excoffier et al., 2013) to estimate the timing of Edge–Margin divergence (*T*_Edge‐Margin_), the timing of Core divergence (*T*_Core_), the ancestral population size (*N*_anc_), the current population sizes (*N*_Core_, *N*_Edge_, *N*_Margin_), and the migration rate between each pair of regions (*m*_Edge‐Margin_, *m*_Core‐Edge_, *m*_Core‐Margin_). The parameters were estimated running 30 independent maximizations of the likelihood based on the observed 3‐dimensional joint SFS derived from the three regional SFS, and retaining the estimate with the highest likelihood. We then performed 100 parametric bootstraps to obtain the 95% confidence interval for each parameter estimate. To avoid making the models overly complex, we modeled changes to *N_e_* as discrete shifts that occurred simultaneously with the timing of population divergence.

Finally, to evaluate the potential impact of non‐neutral sites on the demographic inferences, we reperformed the STAIRWAY PLOT analysis using only the synonymous and intronic sites identified from the variants' annotation. We followed the same procedures and used the same parameters as above. However, because we used only a subsample of sites from the full dataset, and it was not trivial to accurately estimate the total length of the covered genome consisting of just synonymous and intronic sites (*L*), we did not convert coalescent parameter values into absolute estimates of *N_e_* or time. Instead, we compared *θ* per site over time.

## 3 RESULTS

### 3.1 Sequencing and mapping

From the sequencing of the exome bait‐capture libraries, we obtained on average 2.56 million cleaned reads per individual, with a minimum of 1.14 million and a maximum of 4.8 million reads. BWA‐MEM mapping of reads against the reduced white spruce reference resulted in an average of 62% of the reads being mapped with a quality score 20, which were kept for further analyses. The rate of PCR duplicates was approximately 10% for most of the samples (Figure S2), a relatively low proportion in comparison with a similar study on other *Picea* spp. exome capture (Suren et al., 2016). Only one individual (YRB_01) showed more than 50% of PCR duplicates and was subsequently removed from further analysis.

### 3.2 Capture efficiency and polymorphism

**Figure 2:**
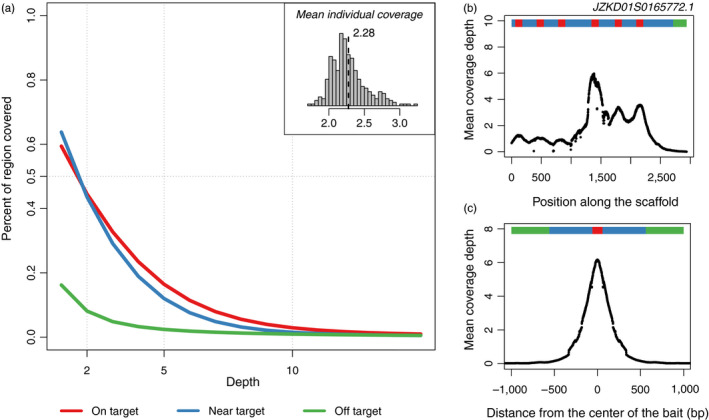
Whole‐exome capture results with the cumulative distribution of coverage for on‐target, near‐target (<500 bp from a bait region), and off‐target (>500 bp from a bait region) loci (a). The mean individual coverage along one scaffold (*JZKD01S0165772.1*) (b) and the mean nucleotide coverage across all scaffolds for on‐target, near‐target, and off‐target regions (c)

After filtering out positions covered by fewer than 15 reads across the complete sampling, the sequences resulting from the exome capture experiment covered a total of 99.7 Mbp in the white spruce‐reduced reference genome. The coverage per individual was 2.28x on average with most individuals showing a mean coverage between 2x and 3x (Figure 2a). We followed the classification of Suren et al., (2016) by labeling the position as “near target” when reads mapped within 500 bp upstream or downstream of a bait edge and off‐target when they mapped outside of this threshold. As expected, coverage was higher for on‐target or near‐target regions, with almost 50% of these regions covered with more than 2 reads on average and across all samples, whereas only 10% of the “off‐target” bases showed the same mean coverage (Figure 2a). The mean coverage across individuals showed peaks of high coverage associated with the targeted regions and a rapid decrease in coverage upstream or downstream of these positions (Figure 2b,c).

Following filtering and coverage thresholds, we kept only sites sequenced in all three regions, resulting in a dataset of 42,328,740 covered bases (i.e., half of the 99.7 Mbp covered by the complete experiment) and 1,372,627 polymorphic sites. For 1,363,244 of these variants (99.3%), we found a reliable correspondence in the Norway spruce genome and were able to annotate them (Table 1). Among the annotated variants, 9.7% were situated within exons consisting of nonsynonymous (6%) and synonymous (3.7%) polymorphisms and 6.7% were situated within introns. Consistent with Suren et al. (2016), we found a large portion of the variants came from regions outside of annotated gene space (70.2%, when Suren et al. (2016), found 60% for a similar experiment in white spruce) or from noncoding regions within 5kb upstream or downstream of exomic regions (6.5% and 6.9%, respectively), despite the design of the exome capture probes based on expressed sequences.

**Table 1:** Summary of genetic variants and their functional annotation identified by mapping the exome capture sequences against the Norway spruce annotated genome

Category	Frequency	Percent
Downstream	94,533	6.9
Nonsynonymous	81,467	6.0
Synonymous	49,967	3.7
Upstream	88,035	6.5
Intron	91,721	6.7
Intergenic	957,521	70.2
Total	1,363,244	100.0

### 3.3 Genetic differentiation

The PCA performed on the 339 individuals pruned dataset (114,699 polymorphic sites) identified three well‐differentiated genetic groups, corresponding to three different genetically and geographically defined regions across the species range: the main northern part of the range (“Core”), a transitional or marginal region in Pennsylvania (“Margin”), and a low latitude trailing edge (“Edge”) region in the southern Appalachians (Figure 1a,b). The first principal component (PC1, 1.6%) differentiated the Margin and Edge regions, while the second principal component (PC2, 0.8%) differentiated the Core from the Margin and Edge (Figure 1b). The PCA also revealed six individuals sampled in two populations of the southern Appalachians (Edge region) that showed a genetic makeup similar to northern (Core region) genotypes despite having been sampled in southern Edge populations. By looking only at these two first PCs, there was no overlap among the three regional genetic groups and the 10 populations from the Margin region showed a great spread along both PC1 and PC2 when compared to within the Edge region.

Population fixation indices were low among the three regions with all pairwise *F*_ST_ values <0.03. The highest estimates were found between Margin and Core (*F*_ST_ = 0.0275) and between Margin and Edge (*F*_ST_ = 0.0273), while the *F*_ST_ value between Core and Edge populations was 0.0229.

### 3.4 Linkage disequilibrium and nucleotide diversity

Considering the signal of genetic structure observed in the PCA, we analyzed the three different genetic regions (Core, Margin, and Edge) independently for the pattern of LD decay, Watterson's *θ*_w_, pairwise nucleotide diversity (π), and Tajima's *D* parameters. The SFS used to infer *θ*_w_ and Tajima's *D* were thus estimated from a genetic dataset considering only sites present in all three regional populations, resulting in a dataset of 42,328,740 covered bases (i.e., half of the 99.7 Mbp covered by the complete experiment) and 1,372,627 polymorphic sites.

**Figure 3:**
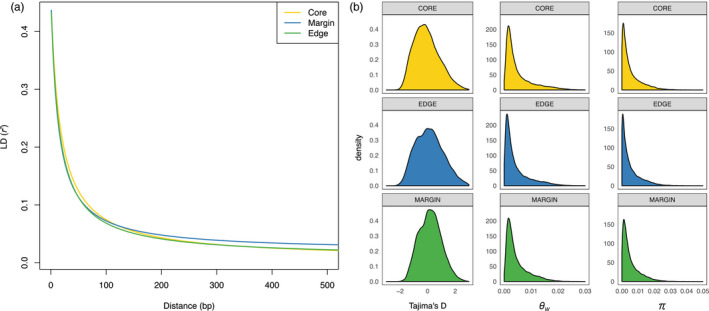
Population genetic parameters of LD decay shown by the curve obtained from the nonlinear regression of *r* ^2^ on physical distance in base pairs (a), and the distribution of Tajima's *D*, Watterson's *θ*_w_, and pairwise nucleotide diversity π across polymorphic sites for each of the three regions (b)

A very low level of linkage disequilibrium was observed among loci (Figure 3a). The mean *r^2^* was .043 (*SD* = 0.093) and LD decayed rapidly, with *r^2^* dropping below .1 within less than 100 bp. The rate of LD decay when estimated independently for the three regions was strongly concordant for Core, Margin, and Edge datasets. The distance at which *r^2^* was half of the initial value was 21, 17, and 18 nucleotides for Core, Margin, and Edge regions, respectively. The per‐region distribution of Tajima's *D* estimates showed value close to 0 for the Core region (−0.06, *SD* = 0.9) and positive values for the Margin (0.19, *SD* = 0.8), and the Edge (0.15, *SD* = 1) regions (Figure 3b), suggestive of a demographic bottleneck for the latter two regions. Nucleotide diversity per site estimated by Watterson's theta (*θ*_w_) and pairwise divergence (π) were also very similar among the three regions (Figure 3b). The Core region showed a mean per‐site *θ*_w_ of 0.0052 (*SD* = 0.0055), while Margin and Edge populations returned a mean per‐site value of 0.0046 (*SD* = 0.0043) and 0.0044 (*SD* = 0.0049), respectively. Similarly, π was 0.005 for Core (*SD* = 0.0063), 0.0054 for Margin (*SD* = 0.0061), and 0.0054 for Edge (*SD* = 0.0061).

### 3.5 Demographic inferences

**Figure 4:**
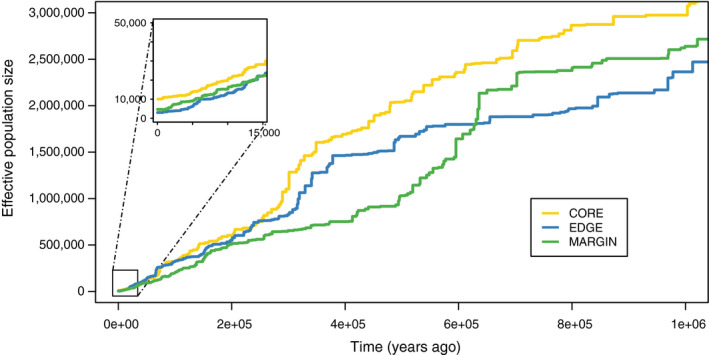
Effective population size (*N*_e_) over the last 800,000 years obtained from the STAIRWAY PLOT method. The inferred *N*_e_ is plotted from present time to the past in years (assuming 29 years per generation). The lines show the median values for each regional genetic group from inferences based on 200 bootstrap samples of the folded SFS without taking the singletons into account

We used the same SFS to infer demographic history as we used to infer *θ*_w_ and Tajima's *D*. STAIRWAY PLOT analyses revealed that red spruce has experienced a strong decline in *N*_e _for hundreds of thousand years (Figure 4). The three regions analyzed separately showed a very similar pattern in the timing and magnitude of *N*_e_ decrease, with strong overlap among regions in their 95% confidence intervals (Figure S4). The decrease in *N*_e _appeared progressive, starting at least 700 thousand years ago (kya) with an acceleration of the decline visible between 600 and 400 kya (Figure 4). During this period, Core, Margin, and Edge ancestral *N*_e_ suffered a reduction from ~2,000,000 to ~700,000 individuals. The *N*_e _of all three regions followed a declining trend until the present day, although the Core *N*_e_ seemed to stabilize in the last 3,000–5,000 years, while the Edge and Margin *N*_e_ continued to decrease toward a lower effective size. Current‐day *N_e_* values suggested 10,039 individuals (upper 95% CI = 152,324) for the Core, 4,655 individuals (upper 95% CI = 39,811) for the Margin, and 3,015 individuals (upper 95% CI = 15,897) for the Edge regions. It is important to note that inferred timing and *N_e_* estimates are directly dependent on the generation time and the mutation rate used for the analyses (respectively 29 years and 2 × 10^–9^ mutation per base and per generation). Hence, *N_e_* estimates would be larger (or smaller) if the true mutation rate was slower (or faster) than assumed.

The TreeMix analysis supported a scenario characterized by an initial split of the Core, followed by divergence between the Edge and Margin regions. TreeMix also supported the occurrence of gene flow between Edge and Core regions, and also between our outgroup (white spruce) and the Margin region (Figure S3), the latter possibly reflecting a history of introgression between red spruce and a related taxon. We used this scenario of divergence events among regions to then estimate the associated parameter values of timing, population size changes, and migration rates using Fastsimcoal2. The current *N*_e_ estimates returned by Fastsimcoal2 were 39,143 (95% CI: 36,357–41,928) for Core, 6,919 (95% CI: 6,527–7,311) for Margin, and 11,812 (95% CI: 11,117–12,506) for Edge, consistent with and within the confidence limits of the STAIRWAY PLOT results. The two events of divergence between the three regions occurred close together in time, both around 9,000 years ago, with an initial divergence of Core population 8,874 years ago (95% CI: 8,344–9,403) and a split between Edge and Margin 8,845 years ago (95% CI: 8,325–9,364) (Table 2), which places each event within the early Holocene. The migration rates estimated among regions were low, with 4.2E‐6 between Core and Margin, 7.9E‐6 between Core and Edge, and 7.5E‐6 between Margin and Edge, roughly corresponding to an exchange of one individual every 4–20 generations. The model also returned a large ancestral effective population size (*N*_ANC_) of 1,036,314 individuals (95% CI: 991,917–1,080,710). This value was consistent with the values of *N*_e_ observed in the STAIRWAY PLOT 300 thousand years ago (Figure 4).

**Table 2:** Demographic parameters inferred with Fastsimcoal2

Parameters	Estimates	95% CI
Divergence times		
*T*_CORE_	8,874	8,344–9,403
*T*_EDGE‐MARGIN_	8,845	8,325–9,364
Effective sizes		
*N*_ANC_	1,036,314	991,917–1,080,710
*N*_MARGIN‐EDGE_	35,957	32,200–39,713
*N*_CORE_	39,143	36,357–41,928
*N*_EDGE_	11,812	11,117–12,506
*N*_MARGIN_	6,919	6,527–7,311
Migration rates		
*m*_CORE‐MARGIN_	4.20E−06	0–1.86E−05
*m*_CORE‐EDGE_	7.90E−06	0–2.14E−05
*m*_EDGE‐MARGIN_	7.50E−06	0–1.44E−05

Note

The values have been translated into effective population size and years assuming a mutation rate of 2 × 10^–9^ and a generation time of 29 years.

When the STAIRWAY PLOT was conducted using only synonymous and intronic sites, the results showed a very similar general trend, with slight variation in effective population size and timing values compared to the analyses on the full dataset (Figure S5). For example, the STAIRWAY PLOT returned the same trend of dramatic decrease in *theta* per site over time with either the full sampling or only the synonymous/intronic sites (Figure S5), but the current *theta* values estimated were higher with only the synonymous/intronic sites than the ones estimated with the complete sampling.

## 4 DISCUSSION

As the impacts of anthropogenic global change accumulate, high‐elevation forest ecosystems are becoming increasingly challenged to migrate, adapt, or endure the stress of a rapidly changing environment (Aitken, Yeaman, Holliday, Wang, & Curtis‐McLane, 2008; Nogués‐Bravo et al., 2018). Impacts from a century and a half of logging and atmospheric pollution have already severely reduced the areal extent of spruce–fir forests in portions of the eastern USA and Canada (White & Cogbill, 1992), and species distribution models predict a further reduction in suitable area under climate change forecasts (Beane & Rentch, 2015; Iverson, Prasad, Matthews, & Peters, 2008). A highly fragmented geographic distribution, coupled with predicted high sensitivity to climate change, poses serious challenges for natural migration in red spruce to keep pace with global warming, especially southern edge populations. Thus, future population viability of this foundation species will be determined less by its potential for natural migration and more by its evolutionary potential to respond to selection in situ*,* or through restoration efforts employing assisted migration aimed at increasing *N*_e_ and/or moving alleles conferring climate adaptation to their future optimal environments (Aitken & Whitlock, 2013; Fitzpatrick & Keller, 2015). Both strategies require a comprehensive understanding of the genome‐wide diversity present within the species, and the spatial context with which this diversity is currently distributed across the landscape.

Our study of genomic diversity in red spruce is the most comprehensive to date, in terms of both genomic coverage and geographic sampling breadth. Our sample covered 99.7 Mbp of exomic sequence representing over 38K expressed genes, and characterized the genomic diversity in a large sample of individuals spanning 65 populations across the entire range, from Atlantic Canada to the southern Appalachian Mountains. From this extensive sample, we have discovered evidence for a long‐term decline in *N*_e_ that appears to have been a pervasive feature of the evolutionary history of red spruce dating back hundreds of thousands of years. Further, genetic subdivision of the species has isolated populations into three regionally divergent groups that show lack of connectivity between the current range Core of the species in the northeast and the fragmented Margin and trailing Edge populations in the southeast. This long‐term decline and currently low *N*_e_ raise questions about whether red spruce populations contain sufficient variability to ensure evolutionary potential to respond to changing selection pressures while avoiding the effects of inbreeding and genetic drift (Hedrick, 2001). Below, we discuss these findings in detail and interpret them in the context of their conservation implications for the adaptive potential and restoration of red spruce in the face of a changing climate.

### 4.1 A history of population decline and genetic subdivision

We used two different but complementary methods to infer contemporary and historical *N*_e_ from the exome‐wide SFS (STAIRWAY PLOT and Fastsimcoal2), and both agreed that red spruce has undergone a precipitous population decline to its current *N_e_* below 20,000 individuals. The first major demographic event recorded by the STAIRWAY PLOT analyses was a strong bottleneck occurring ~400 kya and ending with a loss of 65% of *N*_e_ (from ~2,000,000 to ~700,000 individuals). The timing of this bottleneck in the mid‐Pleistocene is congruent with the timing proposed in the literature for the initial divergence of red spruce from black spruce during the Pleistocene (Jaramillo‐Correa & Bousquet, 2003; Lockwood et al., 2013; Perron, Perry, Andalo, & Bousquet, 2000). This lineage splitting event would likely have resulted in a strong genetic bottleneck, consistent with the findings of other genetic studies that infer red spruce to be a derivative population with a greatly reduced subset of the diversity present in the black spruce progenitor (Jaramillo‐Correa & Bousquet, 2003; Perron et al., 2000). The STAIRWAY PLOT estimate of the postbottleneck *N*_e_ was also consistent with the values for the ancestral *N*_e_ obtained with Fastsimcoal (Table 2), suggesting *N*_e_ represents a loss of 99% of the ancestral population size. The positive Tajima's *D* values for Edge and Margin, estimated independently from the SFS, are also consistent with declining or bottlenecked populations, and confirm the general demographic trend.

Other studies have also reported genetic evidence of demographic decline in red spruce. Jaramillo‐Correa, Gérardi, Beaulieu, Ledig, and Bousquet (2015) used chloroplast SSRs to test for a genetic bottleneck, and found an approximate 50% reduction in chloroplast *N*_e_ occurring approximately 32 kya. Studying red spruce individuals sampled from WV and MD in the Edge region with nuclear SSRs, S. Keller and R. Trott (in prep) found evidence of a more recent bottleneck in *N*_e_ from 7,209 to 535 occurring in the early Holocene, approximately 11,000 years ago. We suggest that these varied estimates, which all indicate a bottleneck in *N*_e_ but with different timing and magnitude, are consistent with the continuous nature of the demographic decline shown by the STAIRWAY PLOT analysis (Figure 4). Hence, variable estimates of *N*_e_ decline likely reflect different datasets and estimators picking up on signals arising at different points along the history of decline, as well as the large stochastic variation arising from sampling and evolutionary processes. The comparison of the STAIRWAY PLOT inference made with the full exomic dataset or with just the synonymous and intronic sites also supports this idea. Here again, the general trend of dramatic decline is unambiguous and confirmed across both datasets, even if the estimations of current *N*_e_ and bottleneck timing differed slightly (Figure S5). Overall, the congruence across multiple different methods, datasets, and samples points to a robust signal of *N*_e_ decline in red spruce, and provides a strong source of cross‐validation needed when making inferences on demographic history from genetic data (Beichman, Huerta‐sanchez, & Lohmueller, 2018).

It is important to note that the approaches we used for demographic inference produce scaled parameter estimates of population diversity and time that can be converted to absolute units of *N*_e_ and years with knowledge of the mutation rate and the generation time, respectively. Mutation rates and generation times are seldom known with precision, and here, we used published values from the literature for mutation rate (2 × 10^–9^) and generation time (29 years) that represent our best available information for red spruce. As a result, while the general trends appear quite robust, the exact timing and magnitude of changes in *N*_e _should be interpreted cautiously. For example, if the true generation time is longer than we assume, then the timing of demographic events we report in this study would be pushed back; likewise, a higher mutation rate would both decrease the value of *N*_e _and move the timing of events forward.

In addition to a history of long‐term decline in *N*_e_ in red spruce, the exome data also suggested genetic subdivision across the range. PCA performed using genotype likelihoods showed a clear genetic structure, with three main clusters that aligned closely with the north–south axis of range fragmentation (Figure 1). The two mostly divergent genetic groups on the PCA differentiated populations of the highly fragmented southern Appalachians (Edge region) from populations from Pennsylvania in the middle of the range (Margin region). The populations from the northern part of the range (Core region) were intermediate along PC1 but were differentiated from Margin and Edge populations along the PC2 axis. However, while PCA clearly revealed genetic structure between different geographic portions of the range, the magnitude of genetic differentiation among regions was relatively weak, with *F*_ST_ values <0.03. Surprisingly, several individuals collected in the southern Appalachians clustered genotypically with populations in the Core region. This probably reflects human planting of northern seed to the south during reforestation efforts of the mid‐20th century.

The higher levels of genotypic diversity (Figure 1) and *N*_e_ (Table 2) for the Core region compared to the Edge region are contrary to expectations of a south–north postglacial recolonization from a single refugium (Holliday, Yuen, Ritland, & Aitken, 2010; Larsson, Källman, Gyllenstrand, & Lascoux, 2013), but it is in line with high northern diversity found in other eastern North American trees species (Lumibao, Hoban, & McLachlan, 2017). In red spruce, the presence of distinct regional genetic clusters, along with variable but not clinal levels of diversity, could suggest the three genetic groups occupied distinct refugia during the last glacial maximum (LGM). Recent studies have found evidence that multiple glacial refugia were involved in the recolonization of several northeastern North American tree species (Mclachlan, Clark, & Manos, 2009; Nadeau et al., 2015). The Fastsimcoal analysis seems to rule out this possibility for red spruce, with a divergence time among regions estimated around 9,000 years ago, well after the ice sheets had retreated and populations would have exited refugia. Another alternative could be that the main glacial refugium for red spruce was close to the ice sheet, as already suggested for other species (Godbout, Beaulieu, & Bousquet, 2010), in the same areas as parts of the current distribution of the Core region genetic cluster. A more specific investigation of the postglacial recolonization routes, for example by combining evolutionary demographic modeling with past species distribution modeling and fossil data, could provide valuable insights into the number and location of glacial refugia for North America forest trees such as red spruce since the LGM (Bemmels, Knowles, & Dick, 2019; He, Prado, & Knowles, 2017).

Another intriguing result was the apparent genotypic variability within the Margin region (Figure 1), despite this group being highly localized geographically. The 51 individuals belonging to the Margin are restricted to a few small populations, but the variation among Margin genotypes was largely responsible for the main genetic gradient present within the dataset (PC1 explaining 1.6% of the genetic PCA). Hybridization is known to increase the genetic variability of introgressed populations (Barrett & Schluter, 2008), and hybridization between black and red spruce has been documented in other studies (de Lafontaine & Bousquet, 2017). One of our study populations in the Margin region (population “XBM”; Bear Meadows Bog, PA) was also sampled in previous studies of hybridization between red and black spruce, and showed clear evidence of mixed genetic ancestry (De Lafontaine, Prunier, Gérardi, & Bousquet, 2015). Thus, one possibility is that the high diversity observed in the Margin region is the result of past or ongoing introgression with black spruce. This hypothesis gains some support from the TreeMix analysis, which inferred migration between the Margin region and our outgroup, white spruce (Figure S3). White and red spruce are not known to hybridize in nature, so we interpret this result as reflective of introgression into red spruce from a related taxon, most plausibly black spruce, which is known to hybridize extensively with red spruce (De Lafontaine et al., 2015). We unfortunately lacked black spruce genomic information in the current study; however, future work will investigate the role for hybridization in shaping genetic variation in the Margin region and in other part of the species' range.

### 4.2 An expanded genomic resource for red spruce conservation

Relatively few genetic studies have been conducted in red spruce, and most are based on a small set of marker loci that have revealed limited genetic diversity, especially compared to its sister taxon black spruce (De Lafontaine et al., 2015; Jaramillo‐Correa & Bousquet, 2003; Perron et al., 2000). Thus, part of our motivation in this study was to expand on the genomic resources available for red spruce. Whole‐exome sequencing is rapidly becoming a preferred technique for population genomic studies of species with large genomes such as conifers (Holliday et al., 2016; Müller et al., 2015; Zhou & Holliday, 2012). Coniferous trees, and especially spruce (*Picea*) species, have received considerable attention with published whole‐exome sequencing datasets for Norway spruce (*P. abies*) (Azaiez et al., 2018), white spruce (*P. glauca*) (Suren et al., 2016), Engelmann spruce (*P. engelmanii*) (Suren et al., 2016), and black spruce (*P. mariana*) (Lenz et al., 2017). With the addition of the whole‐exome data produced for red spruce (*P. rubens*) in this study, four of the six most common spruce species present in North America now have whole‐exome polymorphism data available, opening the door to comparative analyses in this ecologically and economically important genus.

The sensitive part of exome capture experiments is to correctly design the probe set and optimize the efficiency of the capture (Jones & Good, 2016; Neves, Davis, Barbazuk, & Kirst, 2013). In our case, probes were designed based on multiple studies of expressed sequences derived from a variety of tissues sampled in white spruce (Rigault et al., 2011; Yeaman et al., 2014)—a species that diverged from red spruce ~13–20 million years ago (Bouillé & Bousquet, 2005; Lockwood et al., 2013). The availability of a white spruce reference genome is a valuable resource for population genomic studies based on capture sequencing; however, the white spruce genome is huge (>20 Gbp) and highly repetitive, and the assembly is still relatively fragmented (Warren et al., 2015). Despite these challenges, and the relatively large divergence time from red spruce, we obtained good enrichment of target sequences from our exome capture experiment, with 62% of reads properly mapped to the reduced reference genome, covering 99.7 Mbp and allowing the identification of 2,179,980 variants. Sequencing coverage was also greatly centered around the probes (Figure 2), highlighting the advantage of exome capture to target regions within the *Picea* genome most likely to underlie functional variation near expressed sequences, while avoiding wasted sequencing effort on highly repetitive genomic regions. For most of the variants we discovered, we were able to find a corresponding match in the Norway spruce genome, allowing us to functionally annotate the red spruce variant dataset. Knowing the functional class of the polymorphic sites and their potential impact on protein expression allowed us to assess the impact of potentially non‐neutral sites on our demographic inferences, and will be of critical value to future investigations of the signatures of selection and adaptation in red spruce. Surprisingly, a very high proportion of variants were tagged as intergenic sites (>5 kb away from an annotated gene) even though the probes were designed based on expressed sequences (Table 1). A similar pattern of high frequencies of variants annotated as intergenic was found by Suren et al., (2016) for white spruce. In both cases, this could reflect a lack of homology between the white spruce transcriptomic datasets used to design the baits and the Norway spruce reference genome, supporting the need for further improvements to the annotation and assembly of the reference genomes in the *Picea* genus (Suren et al., 2016).

Our study also highlights the benefits of pairing exome capture with low‐coverage sequencing, allowing us to characterize exomes from hundreds of individuals (not pools) sampled from across the species' range at a cost of approximately $110 USD/exome. Given the expected low per‐site coverage, a key component of our strategy was to make use of population genomic pipelines that avoided hard‐calling genotypes and instead used genotype likelihoods to keep the uncertainty associated with each site while maximizing use of all of the data (Fox et al., 2019; Korneliussen et al., 2014; Thorfinn Sand Korneliussen et al., 2013; Meisner & Albrechtsen, 2018). This enabled us to characterize exome‐wide population genomic parameters of nucleotide diversity, LD, and genetic structure while minimizing the introduction of ascertainment biases coming from excessive filtering or genotype misspecification (Nielsen et al., 2011). Use of genotype likelihoods has the added benefit of appropriately handling low probability variants due to sequencing errors intrinsic to next‐generation sequencing. These errors can have tremendous effects on downstream analyses, especially when using the site frequency spectrum (SFS) to infer demographic history or estimate genomic parameters of population growth or selection (Achaz, 2008).

### 4.3 Pattern of LD and its impact on inference of demographic history and adaptation

The level of LD found for red spruce was very low (mean *r^2^* of 0.043 ± 0.09) and showed a rapid decay with physical distance (half‐decrease of LD in 19 bp on average). Rapid decay of LD suggests a historically large mating population, and is consistent with levels of LD found in other coniferous tree species (Jaramillo‐Correa, Verdú, & González‐Martínez, 2010) although the LD decay for red spruce was slightly more rapid than for Norway spruce (Heuertz et al., 2006; Larsson et al., 2013) or white spruce (Pavy, Namroud, Gagnon, Isabel, & Bousquet, 2012). Levels of LD can be population‐specific and influenced by different histories of drift among structured populations (Sawyer et al., 2005); thus, a history of population divergence and admixture could lead to intraspecific divergence in LD (Larsson et al., 2013). In the case of red spruce, strong regional genetic structure (Figure 1b) was accompanied by similar levels of within‐region nucleotide diversity and LD for the three regions. A plausible explanation for rapid and regionally homogenous LD decay is a legacy of high recombination and effective population size in the common ancestor of red spruce, possibly even prior to the split with its sister taxon, black spruce. This would be expected if rates of new mutation were low and the generation time was long—both evolutionary characteristics of *Picea*.

The presence of low LD has implications for the efficacy of genome scans for genes under selection (Kim et al., 2007) and for demographic inference. Weak LD makes it more difficult to identify the selected part of the genome, since the effects of hitchhiking selection will be concentrated very close to the causal variants directly under selection (Myles et al., 2010). For red spruce, and similarly for other species of spruce with low LD, genome scans will thus require very dense genomic data to robustly detect genomic regions under selection. At the same time, low LD also suggests that if signatures of selection are found and truly correspond to regions under selection, they are very likely close to the causal variant. Likewise, rapid LD decay strengthens the inference of demographic history, since a greater proportion of SNPs are acting neutrally and independently, both of which are key assumptions of many demographic analyses (Schrider, Shanku, & Kern, 2016). In support of this view, we found very few qualitative differences in the timing or magnitude of population decline when performing the demographic inferences with either the full exomic dataset or only the synonymous and intronic sites. While the inclusion of non‐neutral polymorphic sites may severely influence demographic inferences (Pouyet, Aeschbacher, Thiéry, & Excoffier, 2018), the large number of variants identified here together with the very rapid decay in LD probably significantly reduced this potential bias in red spruce.

## 5 CONCLUSIONS

Conserving biodiversity requires a good understanding of the factors impacting species genetic variability and evolutionary potential. For red spruce, it seems clear that the recent population dynamics have been strongly influenced by human activities. During the two last centuries, acid rain, logging, and fires have tremendously impacted the survival capacity of the species throughout its range (Mathias & Thomas, 2018; Rentch et al., 2016). With the passage of the Clean Air Act in the USA and restoration efforts employing reforestation and silvicultural practices aimed at red spruce recovery, populations seem poised to recover (Kosiba et al., 2018; Verrico et al., 2019). In the Edge region of the southern and central Appalachians, multi‐agency consortiums have formed with the common goal of restoring a functioning red spruce ecosystem, involving the planting of tens of thousands of trees in order to establish corridors connecting fragmented patches (Central Appalachian Spruce Restoration Initiative and Southern Appalachian Spruce Restoration Initiative). Looking at the particularly small *N_e_* values found in this region, these restoration programs are probably key to preserving red spruce populations in the south of its range, at least at short‐ to medium‐term temporal scales.

However, when looking at a longer timescale, the global trend of the species' demographic history lacks any sign of stabilization or expansion in *N*_e_, even during or in between the different interglacial periods of the late Pleistocene. This sustained decrease in *N*_e_ probably dates back to the very beginning of red spruce as an incipient species. Red spruce is also a relatively young species (Lockwood et al., 2013) and has a restricted and highly fragmented distributional range. A restricted range is often associated with more ecological specialization and a young age means that the species has not yet experienced numerous climatic fluctuations, both factors that can increase the risk of extinction (Liow, 2007; Tanentzap, Igea, Johnston, & Larcombe, 2017; Willis, 1926). However, the species has experienced warmer climatic conditions than most of the other spruce species in North America, especially in the southern part of its range. If these climates have triggered local adaptation, then regional diversity in selected alleles and adaptive phenotypes could be integral to spruce persistence in the context of global climate change. The identification of climate adaptive variation, aided by the availability of the exome sequences reported here and combined with range‐wide provenance trials, could give conservation geneticists and resource managers additional options with which to enhance evolutionary potential within local populations, or to facilitate adaptive gene flow between fragmented populations through assisted migration.

## CONFLICT OF INTERESTS

None declared.

## Supporting information

Figure S1

Figure S2

Figure S3

Figure S4

Figure S5

Table S1

## Data Availability

Sequence data are available in the form of fastq files with the raw reads of each individual in SRA NCBI database (https://www.ncbi.nlm.nih.gov/sra/), deposited as PRJNA625557 bioproject. A vcf file with the genotype likelihoods and annotations and a table with samples information are available on the Dryad repository (http://dx.doi.org/10.5061/dryad.kwh70rz17) (Capblancq et al., 2020). Scripts used to perform the different analyses are available on GitHub (https://github.com/Capblancq/RedSpruce/tree/master/Whole‐Exome‐Sequencing).
