# Supplementary figures and images for: Whole‐exome sequencing reveals a long‐term decline in effective population size of red spruce (Picea rubens)
Source: Evol Appl. 2020 May 22;13(9):2190–205. doi: 10.1111/eva.12985 (PMC7513712; doi:10.1111/eva.12985)

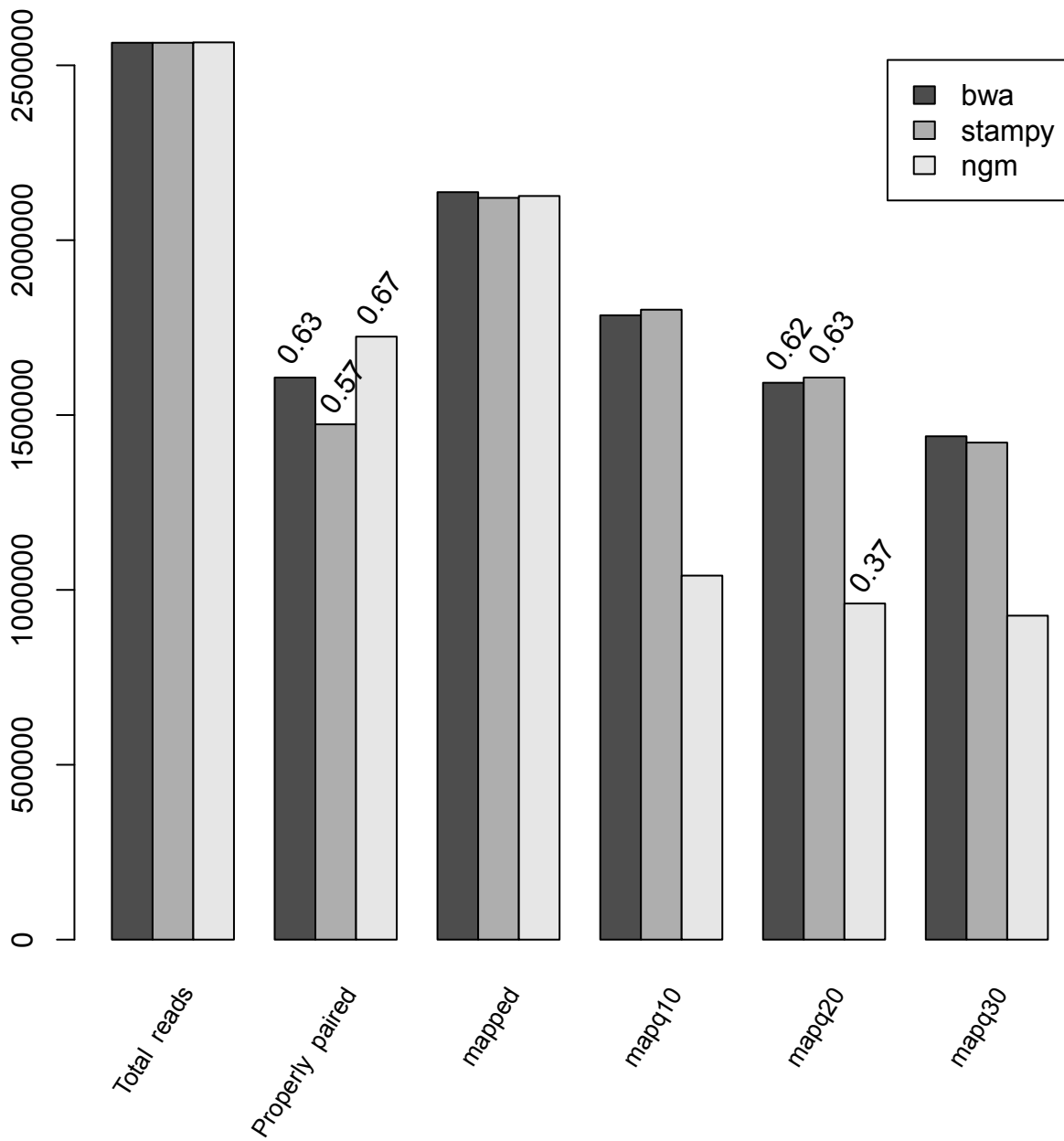

Supplement: Supplementary file 1 — Figure S1 [file EVA-13-2190-s001.pdf]

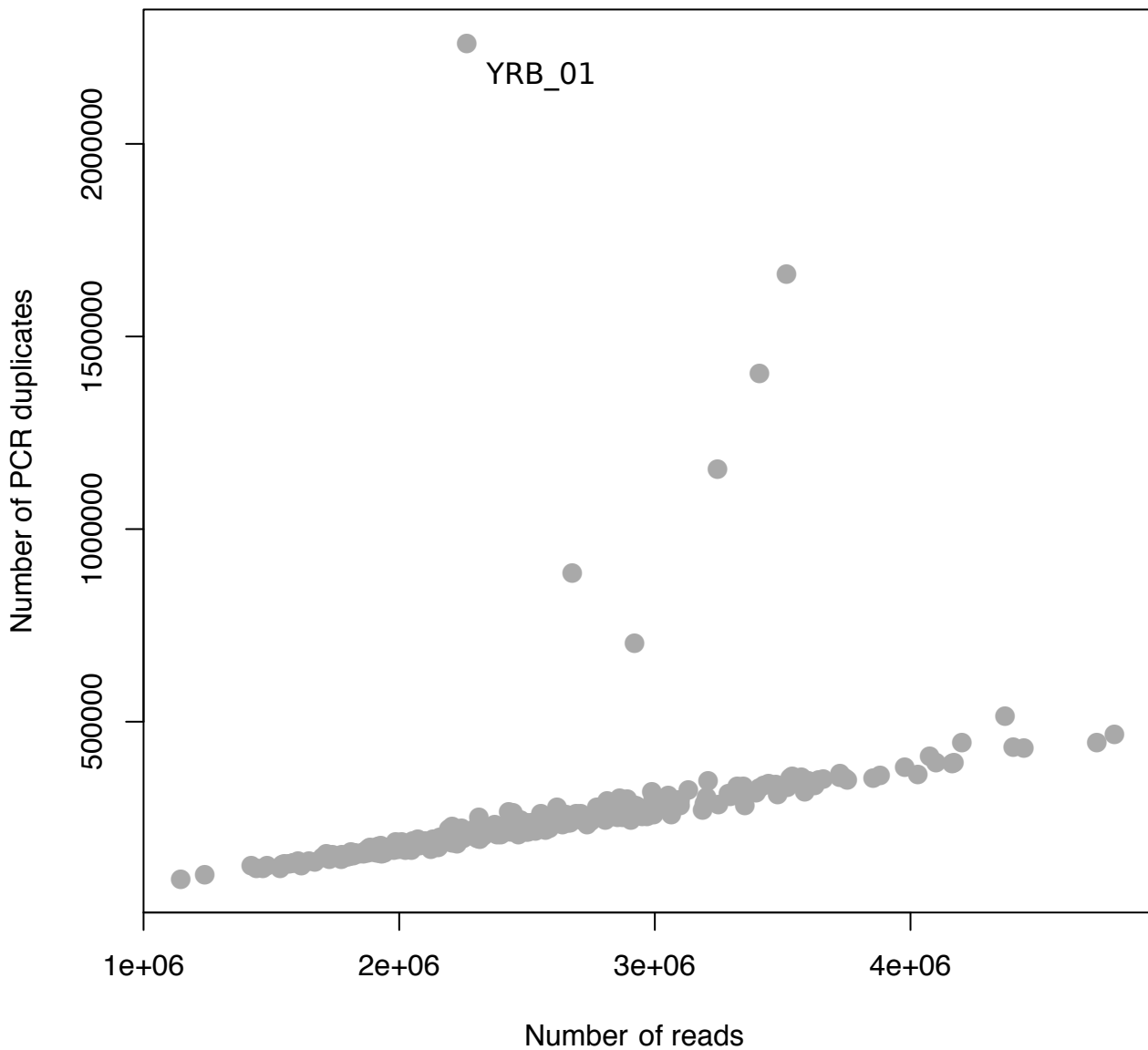

Supplement: Supplementary file 2 — Figure S2 [file EVA-13-2190-s002.pdf]

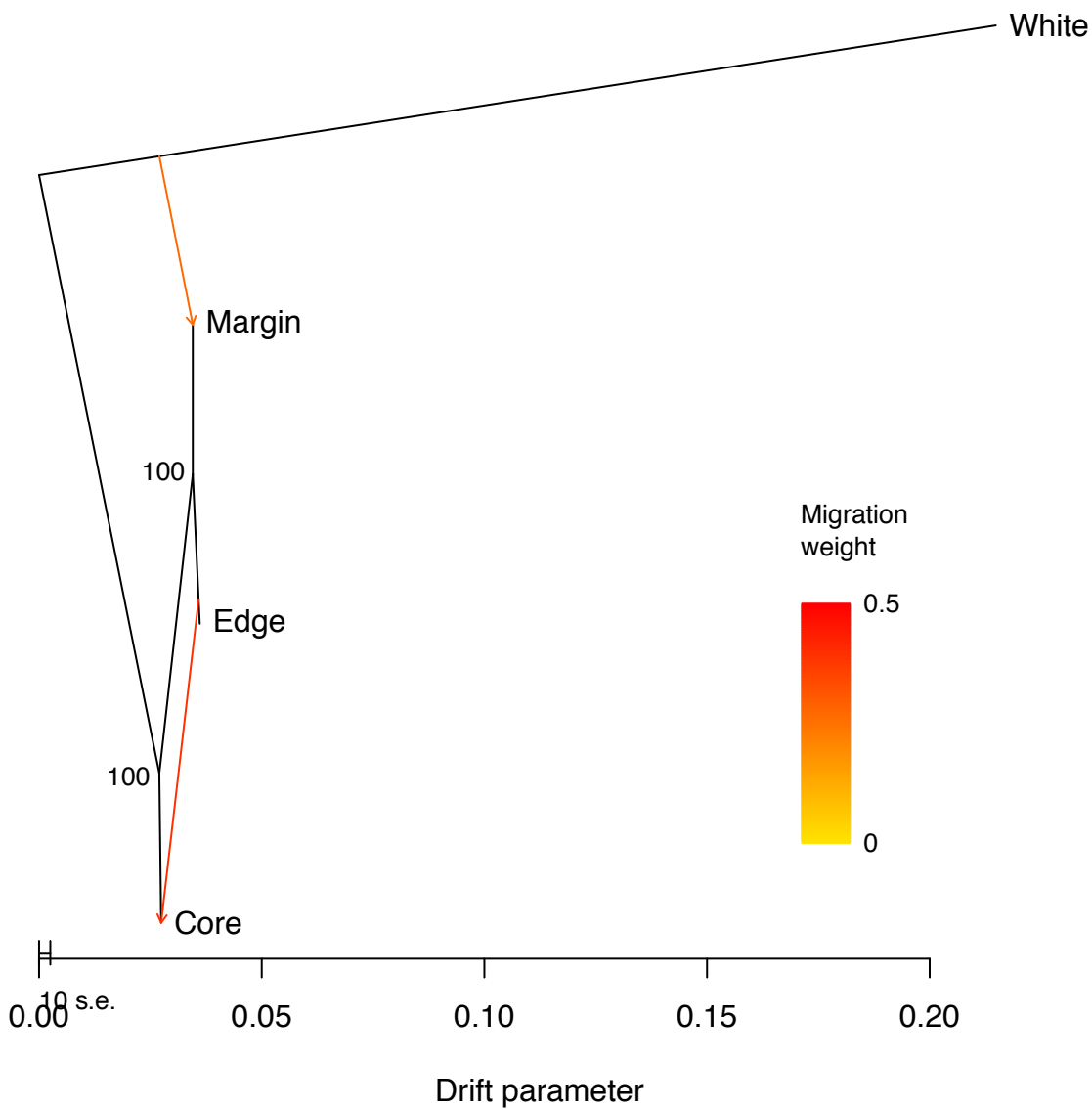

Supplement: Supplementary file 3 — Figure S3 [file EVA-13-2190-s003.pdf]

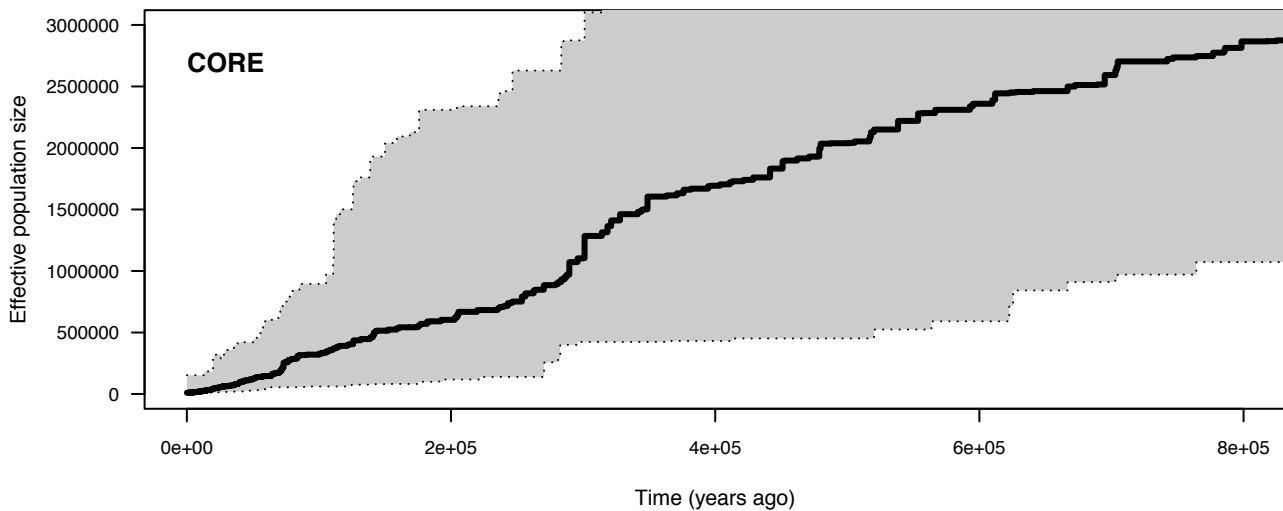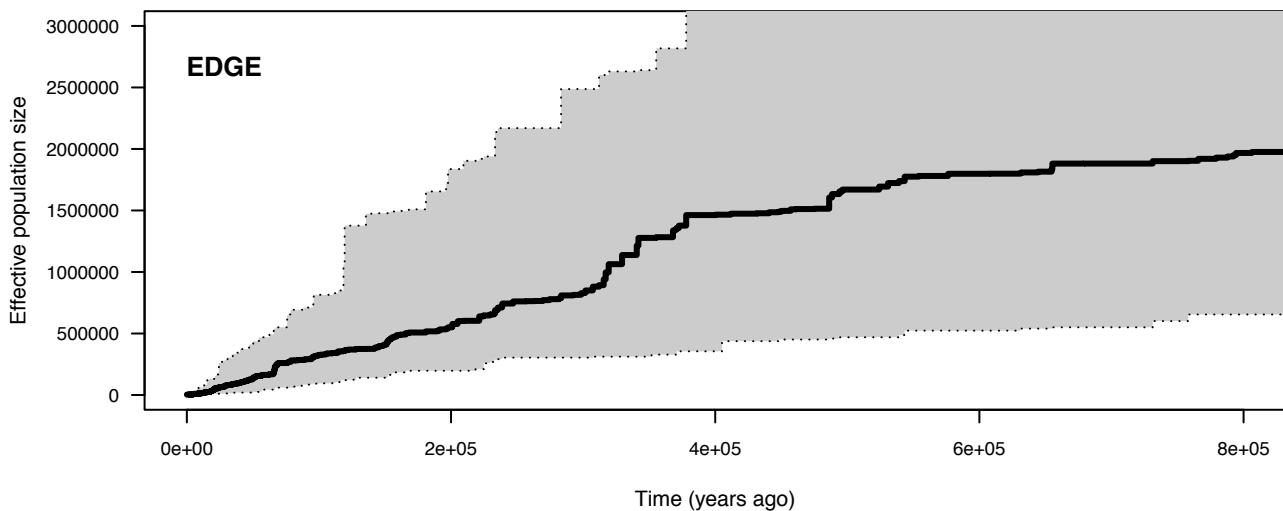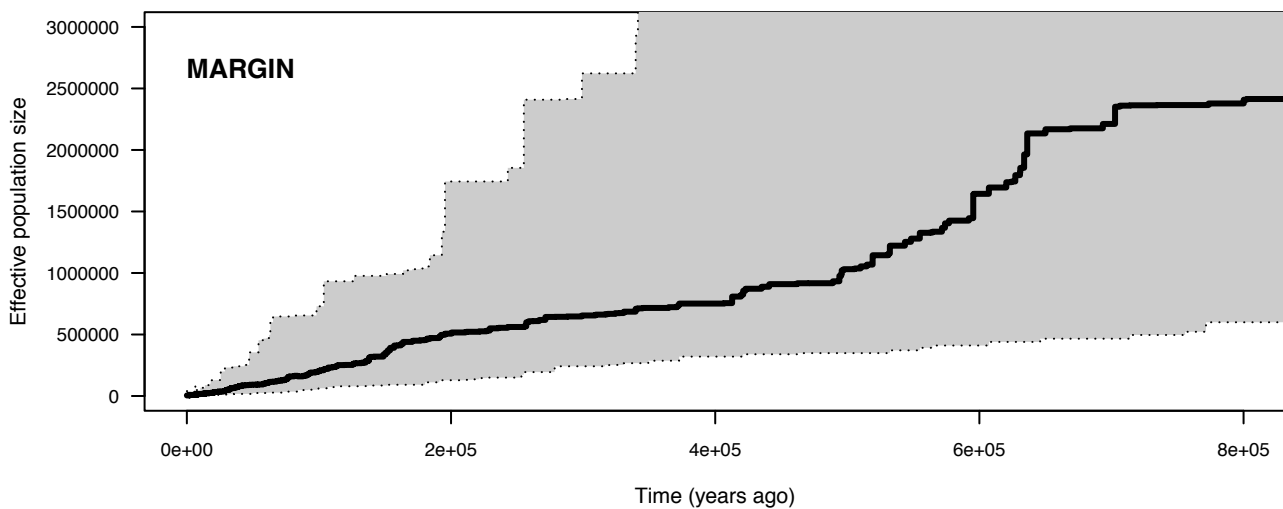

Supplement: Supplementary file 4 — Figure S4 [file EVA-13-2190-s004.pdf]

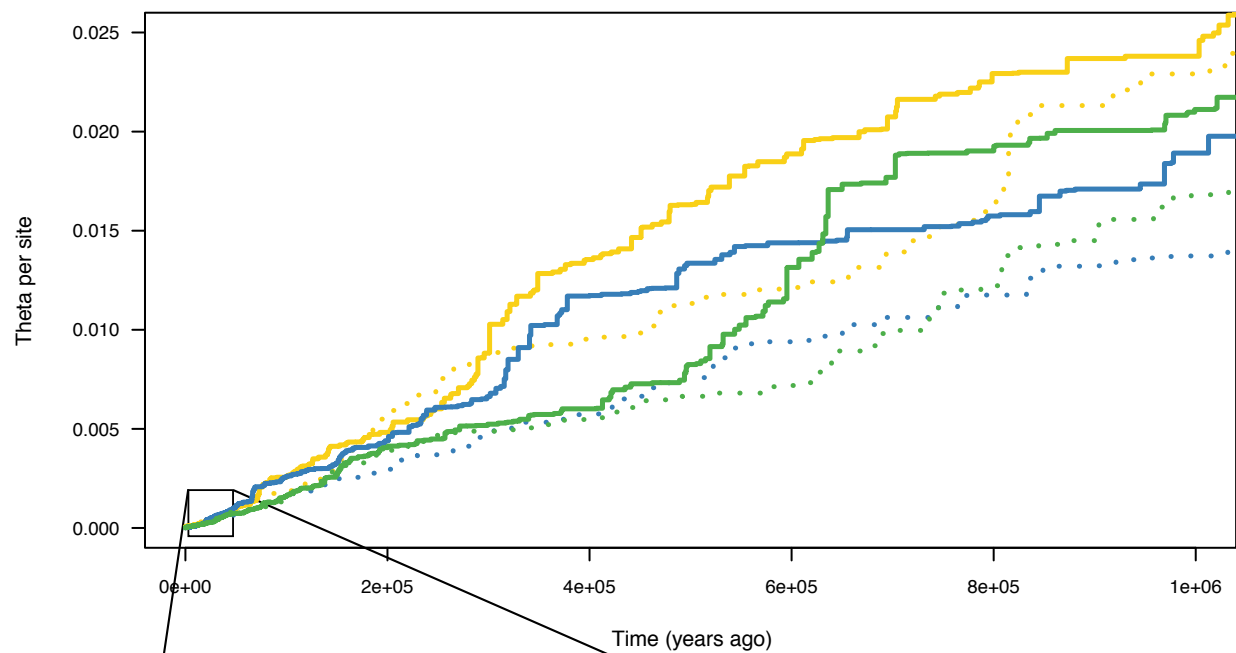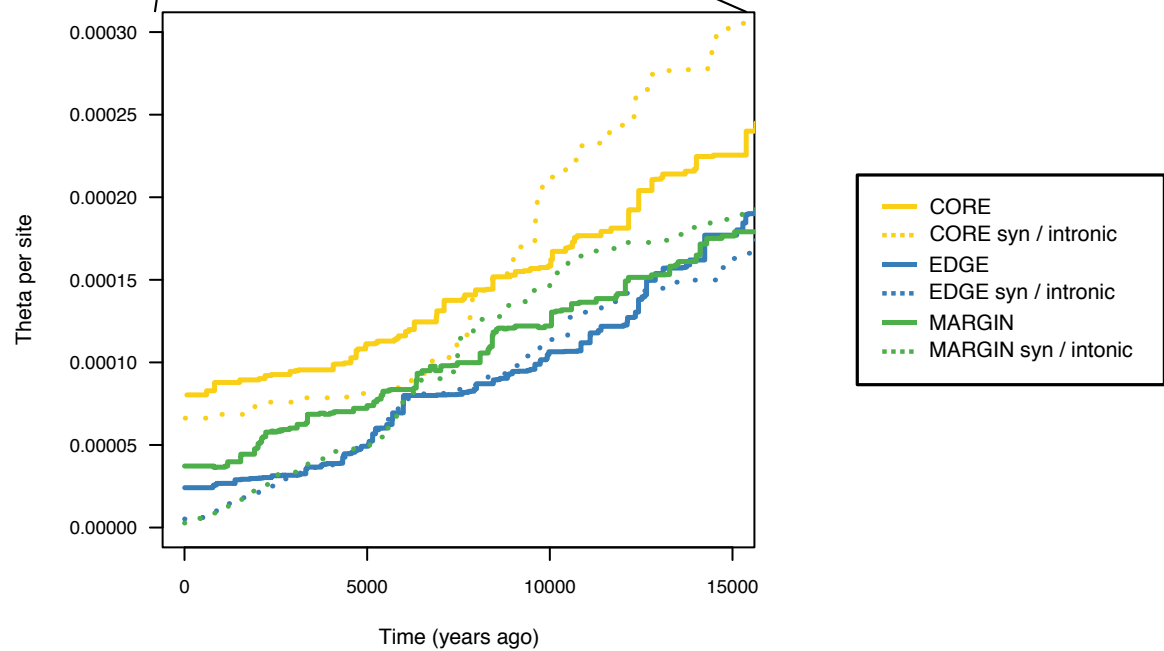

Supplement: Supplementary file 5 — Figure S5 [file EVA-13-2190-s005.pdf]
