# Supplementary material for: Whole‐exome sequencing reveals a long‐term decline in effective population size of red spruce (Picea rubens)
Source: Evol Appl. 2020 May 22;13(9):2190–205. doi: 10.1111/eva.12985 (PMC7513712; doi:10.1111/eva.12985)
Supplement: Supplementary file 6 — Table S1 [file EVA-13-2190-s006.pdf]

Population	Number of samples	State	Region	Latitude	Longitude	Elevation (m)
AB	5	TN	Edge	35.55	83.49	1812
ALB	5	ME	Core	44.31	70.84	455
APP	6	VT	Core	44.21	72.93	743
ASC	6	VT	Core	43.44	72.45	883
B	5	VT	Core	44.11	73.07	311
BAL	6	NY	Core	44.15	73.65	570
BBE	5	NH	Core	43.84	71.56	365
BER	6	ME	Core	44.69	70.38	481
BFA	5	WV	Edge	38.48	79.72	1348
BLA	5	PA	Margin	41.11	75.60	475
BRA	5	PA	Margin	41.16	75.54	520
BRB	4	WV	Edge	39.12	79.58	988
BRU	5	PA	Margin	41.35	75.17	539
CAM	6	VT	Core	44.30	72.91	538
CRR	5	PA	Margin	41.37	76.26	674
CR	6	NC	Edge	35.75	82.27	1819
CRA	2	WV	Edge	38.19	80.27	1072
DG	5	NC	Edge	35.78	82.26	1955
EQU	6	VT	Core	43.14	73.14	608
ESC	6	NY	Core	42.20	74.03	678
G13	5	PA	Margin	41.41	76.30	641
G18	3	PA	Margin	41.10	75.49	538
G57	11	PA	Margin	41.39	76.25	644
GFM	5	NC	Edge	36.09	81.84	1425
GPA	4	WV	Edge	38.62	79.84	1363
GRE	6	MA	Core	42.64	73.17	929
HR	7	TN	Edge	35.57	83.18	1615
HUN	7	NY	Core	42.18	74.23	1221
IND	6	NY	Core	42.17	74.20	646
JAY	3	VT	Core	44.93	72.53	900
KAN	5	NH	Core	44.03	71.50	862
KIL	6	VT	Core	43.61	72.82	1171
KOS	6	WV	Edge	38.69	79.54	1356
LEU	5	ME	Core	45.47	70.37	668
LOL	5	NH	Core	44.14	71.71	865
MAC	5	NY	Core	44.05	73.78	1317
MMF	7	VT	Core	44.52	72.80	887
MPG	6	VT	Core	44.73	72.03	826
MRC	5	VA	Edge	36.67	81.51	1600
MSK	4	NH	Core	44.43	71.43	1197
MT	8	NC	Edge	35.76	82.25	1711
NBTIC	6	NB	Core	46.22	67.14	NA
NOR	6	NY	Core	42.22	74.04	919
OCT	6	MA	Core	42.36	73.16	582
OKE	6	VT	Core	43.41	72.75	812
OWL	4	VT	Core	44.30	72.29	580
PRK	5	WV	Edge	38.33	80.15	1440
PRO	4	VT	Core	44.53	72.87	448
RP	5	TN	Edge	35.61	83.45	1617
SAV	7	MA	Core	42.60	73.03	767
SPR	5	PA	Margin	41.21	75.65	586
TWI	5	NY	Core	42.12	74.13	1064
WA	5	NC	Edge	35.34	83.35	1285
WCA	6	NY	Core	42.47	74.58	574
WHI	6	NY	Core	44.40	73.90	893
XBM	6	PA	Margin	40.73	77.76	552
XCS	2	WV	Edge	39.53	79.48	777
XCV	5	WV	Edge	39.09	79.45	1141
XDS	6	WV	Edge	38.97	79.34	1201
XFS	3	MD	Edge	39.70	78.94	825
XGL	4	MD	Edge	39.51	79.28	815
XPK	4	WV	Edge	38.56	79.50	1340
XSK	3	WV	Edge	38.94	79.69	1127
XWS	3	MD	Edge	39.66	79.09	787
YRB	5	PA	Margin	40.96	75.63	493
